# Bioelectrical impedance analysis for monitoring cancer patients receiving chemotherapy and home parenteral nutrition

**DOI:** 10.1186/s12885-018-4904-6

**Published:** 2018-10-17

**Authors:** Paolo Cotogni, Taira Monge, Maurizio Fadda, Antonella De Francesco

**Affiliations:** 1 Department of Anaesthesia and Intensive Care, Pain Management and Palliative Care, S. Giovanni Battista Hospital, University of Turin, C.so Bramante 88, 10126 Turin, Italy; 2 Unit of Parenteral Nutrition in Oncology, S. Giovanni Battista Hospital, C.so Bramante 88, 10126 Turin, Italy; 3 Clinical Nutrition, S. Giovanni Battista Hospital, C.so Bramante 88, 10126 Turin, Italy

**Keywords:** Cancer, Bioelectrical impedance analysis, Body composition, Malnutrition, Home parenteral nutrition

## Abstract

**Background:**

Home parenteral nutrition (HPN) can improve survival, quality of life, nutritional and functional status in cancer patients. Bioelectrical impedance analysis (BIA) is a non-invasive, validated method to assess body composition. The objective of this prospective single-arm study was to investigate the impact of HPN in advanced cancer patients receiving chemotherapy assessed by BIA, clinical and laboratory measures.

**Methods:**

Adult malnourished cancer outpatients with solid tumors receiving anticancer treatments who were candidates for daily HPN were enrolled. Patients were assessed at baseline (T0), 60 (T1) and 90 days (T2) after HPN start. Assessments included anthropometric and clinical-oncological characteristics, performance status, inflammatory response and Patient-Generated Subjective Global Assessment (PG-SGA).

**Results:**

Sixty-five advanced cancer patients were enrolled. Median overall survival was 317 days. Body weight, BMI, oral calorie and protein intake increased over time (*P* < 0.01). At T2 the proportion of well-nourished patients, Karnofsky performance status and modified Glasgow prognostic score were improved (*P* < 0.01), total body water was reduced (*P* = 0.04), and fat mass increased (*P* = 0.04). Reactance, resistance and phase angle were significantly associated with survival at T0, T1, and T2, respectively. At T2, PG-SGA category A was a predictor of survival (*P* < 0.0001).

**Conclusions:**

After 90 days of HPN, patients experienced significantly improved nutritional status, performance status, prognostic score and some BIA measures. HPN may be an important therapy in oncology patients receiving chemotherapy. Longitudinal use of BIA may help track the effects of HPN and disease progression, potentially contributing to optimal global patient management.

## Background

Malnutrition is commonly reported in cancer patients and can increase mortality, morbidity and treatment complications, and can reduce treatment effectiveness and quality of life (QoL) [1, 2]. Cancer patients are usually at risk of developing cachexia, which is associated with progressive weight loss, loss of muscle and fat tissue, impaired immune function, and metabolic disturbances caused by a variable combination of reduced food intake, systemic inflammation and abnormal metabolism [3, 4]. Chemotherapy can cause side effects that further compromise nutritional status including nausea, vomiting, anorexia, and loss of body weight. Many studies clearly demonstrated that muscle loss strongly predicts the development of chemotherapy toxicity [5, 6]. Early nutritional intervention is critical to optimal patient outcomes [7], yet most patients do not receive intense nutritional therapy until they experience significant weight loss [8–10].

Parenteral nutrition (PN) has been shown to improve survival, QoL, and nutritional and functional status, and slow progressive weight loss in advanced cancer patients [11–18]. Current guidelines recommend home parenteral nutrition (HPN) for cancer patients with chronic defects of dietary intake or absorption when enteral nutrition is not sufficient or feasible, if they are expected to die from starvation prior to cancer spread [19–21].

Clinicians must identify patients likely to benefit from PN, and balance the potential to improve nutritional status, QoL and survival with PN without prolonging life in patients with no chance of improvement [20, 22]. Some key clinical measures may not accurately reflect patient status due to inflammation and disease state. Therefore, clinicians must rely on multiple characteristics such as Karnofsky performance status (KPS), tumor site and spread, and Glasgow prognostic score (GPS) in addition to anthropometric measures to assess patient status and prognosis to guide nutrition management [23, 24].

Cancer patients experience not only loss of overall body weight but also loss of muscle tissue and body cell mass (BCM) [25], and changes in fluid distribution with extracellular expansion and reduced intracellular water. Managing nutritional needs based only on body weight can be misleading, as it does not reflect body composition [26, 27].

Bioelectrical impedance analysis (BIA) is non-invasive, validated method to assess body composition and nutritional status, which offers an efficacious way to track changes in body composition over time [28]. Bioelectrical impedance vector analysis (BIVA) is a pattern analysis of impedance measurements (resistance and reactance) plotted as a vector in a coordinate system [29] that can be used as a quality control measure for interpretation of BIA results. It is useful in assessing hydration status, which is difficult to determine based on biochemical methods and physical exam [30].

BIA assessments such as phase angle (PA) and fat free mass (FFM) have been correlated with nutritional status and survival in cancer patients [26, 31–34]. However, few published studies have examined the utility of body composition obtained from BIA to monitor the effects of nutritional support in cancer patients on HPN [15, 17].

The primary objective of the current study was to investigate the impact of HPN in advanced cancer patients receiving chemotherapy using BIA measures along with clinical and laboratory measures by a prospective study without a control group. The secondary objective was to evaluate the association between BIA measures and other clinical and laboratory variables with survival.

## Methods

### Study design

This prospective, longitudinal study was conducted in a 1200-bed tertiary university hospital. From October 1, 2014 through March 31, 2015, all adult cancer outpatients with solid tumors receiving anticancer treatments (neoadjuvant/adjuvant or palliative chemotherapy with or without radiation therapy) who were candidates for daily HPN based on European guidelines were eligible for enrollment. Criteria for HPN included: proven and prolonged failure to meet nutrition requirements by oral or enteral route, with impending risk of death due to malnutrition; life expectancy > 2 months; KPS > 50; control of pain; absence of severe organ dysfunctions; written informed consent confirming that the patient accepted this modality of nutrition support; approval by the physician responsible for HPN, the oncologist and the general practitioner; presence of environmental conditions compatible with HPN; availability of an in-home caregiver; and availability of a specifically trained nursing team dedicated to the patient home care, as provided by the Public Health Service [35]. Ethics Committee approval was obtained prior to study start, and written informed consent was obtained from each patient prior to any study procedures.

Patients were assessed in a hospital setting by the physician responsible for HPN and the dietician at baseline (T0), at 60 days (T1), and 90 days (T2) after the start of HPN. Data recorded included anthropometric (actual body weight, weight loss, body mass index [BMI]) and clinical-oncological (tumor site and stage, anticancer treatments) assessments. Performance status was graded using the WHO/Eastern Cooperative Oncology Group (ECOG) scale (0 = normal performance, 4 = bed-bound) [36] and KPS (0–100) [37]. Systemic inflammation was estimated using serum C-reactive protein (CRP) and albumin, and inflammatory response was graded according to the modified Glasgow Prognostic Score (mGPS) (0–2) that is highly predictive of morbidity and mortality in cancer patients [38]. At baseline (T0), oral intake was assessed quantitatively using a 24-h food recall by the dietician through a structured interview in which patients were asked to recall all food and drink they consumed in the previous 24 h. Nutrition was assessed using the Patient-Generated Subjective Global Assessment (PG-SGA), which combines qualitative and semi-quantitative data to yield a comprehensive ‘malnutrition score’ (A = well-nourished; B = suspected malnutrition or moderate malnutrition; or C = severely malnourished) [39]. The PG-SGA was developed specifically for patients with cancer and it includes questions regarding the presence of nutritional symptoms and short-term weight loss. It was designed so that the components of the medical history can be completed by the patient using a check box format. In particular, the questions regard current weight, weight history, and acute weight changes; any changes in food intake over the past month; any nutrition impact symptoms experienced over the previous 2 weeks; and any changes to activities and functions over the previous month. Conversely, the physical examination is performed by a physician or dietitian. Start date, end date, and management of HPN were recorded over time.

After the start of HPN, all patients were closely monitored by the physician responsible for HPN through regularly scheduled and structured telephone interviews (at least every 15 days) and home visits by the nursing team and general practitioner (initially daily for 2–3 weeks, and at least every 7 days thereafter). After adequate training, home caregivers administered HPN. HPN was delivered overnight for 10–14 h per day, using standard commercially manufactured ready to use bags containing amino acids, electrolytes, glucose, and lipids. HPN was prescribed to provide 20–25 kcal/kg/day for bedridden or 25–30 kcal/kg/day for ambulatory patients and 1–1.5 g/kg/day amino acid. Every 30 days from HPN start (± 5 days), an outpatient re-evaluation by both the physician and the dietitian (including a 24-h food recall) was performed. Overall survival was calculated as the number of days between the date of HPN start and the date of patient death from any cause, with censoring at the date of last follow-up assessment in alive subjects (at November 30, 2016).

### Bioelectrical impedance analysis

Tetrapolar single frequency BIA is performed by passing current between two surface electrodes placed on the right hand and right foot [40]. The primary carriers of current in tissue are charged ions, such as sodium or potassium therefore conductivity of blood and urine is high, muscle is intermediate, and bone, fat, and air is low. Cell membranes form barriers to the flow of charge, therefore intact cell membranes cause increased resistance to flow [41]. BIA actually measures the voltage (V) produced between two electrodes located near to, but different from, the sites where current is introduced in most cases. The measurement normally is called impedance (Z), which is made up of resistance (RZ) and reactance (XC). Resistance reflects opposition to current due to body fluids and is therefore a measure of cellular hydration, and reactance reflects opposition due to cell membranes and tissues, and indicates cellular integrity. PA is calculated as the arctangent of the ratio of reactance divided by resistance. PA is considered a measurement of overall health [30] and decreases over time due to age, disease, and sedentary lifestyle. BIA does not directly measure total body water (TBW), but rather a weighted sum of extracellular water (ECW) and intracellular water (ICW) resistivity, which allows FFM and TBW to be estimated.

All BIAs were performed by the same dietitian using an impedance plethysmograph that emits an 800-A, 50-kHz alternating current (STA-BIA 50-kHz, Akern, Florence, Italy). BIAs were performed with an empty bladder, after fasting and not receiving HPN for at least 8 h, with patient lying supine on a bed with legs apart and arms not touching the torso. The four standard electrodes were positioned on the ulnar aspect of the right wrist and the right medial malleolus, according to the standardized technique [41].

BIA was performed at T0, T1, and T2. The following parameters of BIA were evaluated: RZ, XC, PA, ECW, ICW, TBW, FFM, fat mass (FM), BCM, extracellular mass (ECM), ECM/BCM ratio, and muscle mass. BIVA was plotted at each time point.

### Statistical analysis

Statistical analysis calculated changes in patient and BIA measures over time. Results for continuous variables were expressed as mean, median and range, and categorical variable as counts and percentage. For variables assessed at T0, T1, and T2, the values for a single variable were plotted per patient, a line was fitted, and the slope was calculated. A t-test was performed to determine whether the mean slope was significantly different from 0 for each variable. Variables assessed at T0 and T2 were analyzed with paired Student’s t-test, and categorical variables were analyzed with Wilcoxon test for paired sample and Mann-Whitney test for independent samples. Survival data were summarized by computing the overall survival curve with the Kaplan–Meier method. The comparison of survival between curves was assessed by log-rank and Wilcoxon test. Hazard ratio (HR) with 95% confidence interval (CI) was also calculated for survival data. Assessment of patient and BIA variables associated with survival was performed using Cox proportional-hazards regression. The accuracy of a test to discriminate diseased cases from normal cases was evaluated using Receiver Operating Characteristic (ROC) curve analysis. A two-sided *P*-value of 0.05 or less was considered significant. All analyses were performed using SPSS Statistics Release 23.0.0 (SPSS, Inc., an IBM Company, Chicago, IL).

## Results

### Patients

Sixty-five malnourished stage III and IV adult cancer patients were consecutively enrolled in the study. Thirty-two (49%) were female, and median age was 64 years (range 33–79). Tumor locations across the population were stomach: 26 (40%), pancreas/biliary system: 15 (23%), colon/rectum: 9 (14%), gynecological: 5 (8%), esophagus: 3 (4%), and other: 7 (11%). Forty-six patients (71%) had metastasis, and 16 (25%) were diagnosed with stage III cancer, and 49 (75%) were diagnosed with stage IV cancer. All patients were receiving chemotherapy (neoadjuvant: 9, adjuvant: 37, and palliative; 19, respectively) and 10 received both chemotherapy and radiation therapy. Adjuvant chemotherapy was given in 37 patients following surgery, while neoadjuvant chemotherapy in 5 patients that subsequently underwent surgery. Overall, 42 patients (65%) received surgery. Median percent weight loss in the 3 months prior to the start of HPN was 10.7% (range 1.9–27.8%). At baseline (T0), all patients had residual but insufficient oral food intake (a median of 600 Kcal), and supplemental HPN provided a median of 1200 and 1000 kcal per day at T1 and T2 respectively.

No HPN-related metabolic complications occurred. The incidence of catheter-related bloodstream infections (CRBSI) was 0.27 *per* 1000 catheter-days. The overall rate of catheter-related complications (i.e., mechanical, CRBSI, and venous thrombosis) was 1.06 *per* 1000 catheter-days. No patients required hospitalization due to complications.

### Clinical and BIA measures

Table 1 shows patient and BIA variables at T0, T1, T2, and the calculated mean slopes for variables with assessments at all three time points. Actual body weight and BMI, as well as oral calorie and protein intake had significantly positive slopes, and the proportion of patients with BMI < 18.5% decreased from 33.8 to 30.7% from baseline to T2. At T2 a greater proportion of patients qualified as PG-SGA category A (well-nourished), and KPS and mGPS were significantly improved. The proportion of patients with KPS > 70 increased from 66.2 to 77% at from baseline to T2. Among BIA variables, TBW had a significantly negative slope, and FM had a significantly positive slope from baseline to T2.

**Table 1:** Changes in patient and BIA variables over time

	T0	T1	T2	Mean Slope (change per day)	*P*
Patient Variables					
Actual body weight, kg	57.4 [57.0] (33.0–100.0)	58.9 [57.0] (37.7–96.0)	59.7 [60.0] (37.5–95.0)	0.02	**< 0.01**
Body Mass Index, kg/m^2^	21.2 [21.1] (12.7–39.1)	21.7 [21.4] (15.4–37.5)	22.0 [21.7] (15.4–37.1)	0.01	**< 0.01**
Oral calorie intake, kCal	597 [600] (0–1500)	904 [1000] (0–2550)	959 [1000] (0–2550)	4.18	**< 0.01**
Oral protein intake, g/day	21.1 [20.0] (0–50)	32.2 [35.0] (0–110)	34.5 [40.0] (0–90)	0.15	**< 0.01**
PG-SGA category, %	A: 0%	NA	A:8%	NA	**< 0.01**
	B: 48%		B:48%		
	C: 52%		C:44%		
Karnofsky PS	67.4 [70.0] (50–90)	NA	72.5 [70.0] (50–90)	NA	**< 0.01**
ECOG PS	1.3 [1] (0–2)	NA	1.2 [1.0] (0–2)	NA	0.09
Albumin, g/dL	3.4 [3.5] (1.9–4.5)	NA	3.5 [3.5] (2.2–4.6)	NA	0.19
C-reactive protein, mg/L	16.7 [10.9] (0.4–73.2)	NA	13.5 [6.9] (0.1–140)	NA	0.23
mGPS	1 [1] (0–2)	NA	0.6 [0] (0–2)		**0.01**
	0: 37%		0: 62%		
	1: 26%		1: 14%		
	2: 37%		2: 25%		
BIA Variables					
Resistance, Ohm	590.9 [606.0] (401–804)	586.6 [586.0] (401–898)	584.0 [607.0] (372–780)	−0.08	0.45
Reactance, Ohm	52.0 [48.0] (19–120)	51.0 [49.0] (20–99)	51.2 [46.0] (22–116)	− 0.01	0.68
Phase Angle, °	4.8 [4.8] (2.5–8.1)	5.0 [4.7] (2.5–11.4)	5.0 [4.5] (2.3–10.0)	0.00	0.32
Total Body Water, %	59.4 [59.7] (39.3–85.8)	58.6 [58.5] (41.3–76.6)	58.0 [58.2] (41.7–76.4)	−0.02	**0.04**
Extracellular Water, %,	52.1 [52.2] (25.4–70.7)	52.1 [52.6] (29.5–69.9)	52.2 [53.5] (32.3–73.2)	0.00	0.98
Intracellular water, %	47.9 [47.8] (29.3–74.6)	48.1 [47.7] (30.1–70.5)	47.7 [46.3] (26.8–67.7)	0.00	0.89
Fat Mass, %	23.3 [23.2] (−7.1–50.8)	24.1 [24.6] (0.5–48.4)	25.8 [24.3] (1.6–76.9)	0.03	**0.04**
Fat Free Mass, %	74.6 [76.6] (49.2–86.9)	76.0 [75.4] (51.6–101.2)	75.0 [75.7] (52.5–98.4)	0.01	0.52
Muscle Mass, %	44.0 [39.2] (25.3–75.4)	43.2 [40.0] (19.5–120.7)	42.1 [38.9] (23.5–81.3)	− 0.02	0.37

All values are expressed as mean [median] (range), or percentage as indicated

T0: at start of HPN; T1: 60 days after the start of HPN; T2: 90 days after the start of HPN

*NA* Not applicable, *BIA* Bioelectrical impedance analysis, *BMI* Body mass index, *ECM* Extracellular mass, *BCM* Body cell mass, *PS* Performance status, *ECOG* Eastern cooperative oncology group, *mGPS* Modified Glasgow prognostic score, *PG-SGA* Patient-generated subjective global assessment; Category A = well-nourished; Category B = suspected malnutrition or moderate malnutrition; Category C = severely malnourished

Table 2 shows patient and BIA variables subsetted by PG-SGA at T0 (B or C; entry criteria prohibited category A). In both cohorts, actual body weight, BMI, oral calorie and protein intake had significantly positive slopes, but the positive slopes trended towards being steeper in the group with PG-SGA of B at study entry. There were multiple variables with significant changes in the group with PG-SGA of B at study entry, but not PG-SGA of C at entry: increase in KPS and albumin, decrease in ECOG, decrease in TBW, and increase in FM.

**Table 2:** Changes in patient and BIA variables over time by PG-SGA at T0

	PG-SGA at T0	T0	T1	T2	Mean Slope (change per day)	*P*
Patient Variables						
Actual body weight, kg	B	60.2 [57.5] (39.0–100.0)	62.3 [59.6] (41.5–96.0)	62.8 [60.0] (43.5–95.0)	0.03	**0.01**
	C	54.9 [56.5] (33.0–85.5)	55.7 [56.4] (37.7–83.0)	56.8 [58.5] (37.5–80.0)	0.02	**0.02**
Body Mass Index, kg/m^2^	B	22.0 [21.2] (14.5–39.1)	22.8 [21.9] (16.5–37.5)	23.0 [22.1] (17.2–37.1)	0.01	**< 0.01**
	C	20.4 [20.7] (12.7–29.1)	20.8 [20.6] (15.4–28.1)	21.2 [20.7] (15.4–29.7)	0.01	**0.02**
Oral calorie intake, kCal	B	744.7 [800] (0–1500)	1190.5 [1200] (0–2550)	1204.2 [1280] (0–2550)	5.44	**< 0.01**
	C	462.4 [500] (0–1300)	642.1 [600] (0–1700)	735.3 [600] (0–1900)	3.03	**< 0.01**
Oral protein intake, g/day	B	24.8 [25] (0–50)	40.6 [40] (0–110)	40.8 [45] (0–90)	0.19	**< 0.01**
	C	17.6 [17.5] (0–50)	24.6 [25] (0–75)	28.8 [30] (0–60)	0.12	**< 0.01**
Karnofsky PS	B	67.7 [70] (50–90)	NA	74.5 [80] (50–90)	NA	**< 0.01**
	C	67.1 [70] (50–80)	NA	70.6 [70] (50–90)	NA	0.1
ECOG PS	B	1.3 [1] (0–2)	NA	1.0 [1] (0–2)	NA	**< 0.01**
	C	1.4 [1] (1–2)	NA	1.3 [1] (0–2)	NA	0.82
Albumin, g/dL	B	3.4 [3.5] (1.9–4.5)	NA	3.7 [3.7] (2.8–4.4)	NA	**0.02**
	C	3.4 [3.5] (1.9–4.4)	NA	3.4 [3.3] (2.2–4.6)	NA	0.70
C-reactive protein, mg/L	B	17.7 [10.5] (0.4–71)	NA	17.3 [4.0] (0.1–140)	NA	0.41
	C	15.7 [12.2] (0.9–73.2)	NA	10.1 [7.2] (0.1–56.5)	NA	0.11
mGPS	B	1 [1] (0–2)	NA	0.6 [0] (0–2)	NA	0.07
	C	1 [1] (0–2)	NA	0.6 [0] (0–2)	NA	0.10
BIA Variables						
Resistance, Ohm	B	560.0 [550.0] (401–720)	573.5 [586.0] (401–722)	567.3 [602.0] (407–698)	0.10	0.42
	C	619.0 [625] (402–804)	598.4 [594] (405–898)	599.1 [610.0] (372–780)	−0.24	0.12
Reactance, Ohm	B	45.4 [42.0] (19–118)	46.2 [42.6] (20–69)	44.8 [42.0] (23–72)	0.00	0.88
	C	58.0 [53.0] (23–120)	55.5 [52.0] (25–99)	57.1 [51.5] (22–116)	− 0.01	0.69
Phase Angle, °	B	4.5 [4.2] (2.5–7.5)	4.6 [4.5] (2.5–6.2)	4.5 [4.4] (2.3–6.3)	0.00	0.82
	C	4.8 [4.8] (2.5–8.1)	5.0 [4.7] (2.5–11.4)	5.0 [4.5] (2.3–10.0)	0.00	0.30
Total Body Water, %	B	59.5 [59.5] (39.3–76.6)	57.2 [58.1] (41.3–72.8)	56.9 [56.5] (42.0–72.0)	−0.03	**< 0.01**
	C	59.4 [60.1] (40.3–85.8)	59.8 [60.0] (43.7–76.6)	58.9 [59.0] (41.7–76.4)	0.00	0.74
Extracellular Water, %,	B	54.1 [56.1] (25.4–70.7)	53.8 [53.8] (44.9–69.9)	54.4 [54.4] (44.2–73.2)	0.00	0.89
	C	50.4 [49.1] (31.7–68.5)	50.5 [50.5] (29.5–69.2)	50.2 [51.5] (32.3–71.3)	0.00	0.93
Intracellular water, %	B	45.9 [43.9] (29.3–74.6)	46.6 [46.3] (30.1–55.1)	45.6 [45.6] (26.8–55.8)	0.00	0.95
	C	49.6 [51.0] (31.5–68.3)	49.5 [49.6] (30.8–70.5)	49.5 [48.0] (28.7–67.7)	0.00	0.89
Fat Mass, %	B	23.8 [25.4] (4.6–50.8)	26.2 [26.9] (0.5–48.4)	28.5 [29.1] (1.6–76.9)	0.05	**0.02**
	C	22.8 [22.7] (−7.3–45.0)	22.1 [20.5] (1.2–45.4)	23.4 [21.6] (3.3–43.0)	0.00	0.78
Fat Free Mass, %	B	74.4 [74.5] (49.2–86.9)	73.8 [73.1] (51.6–99.5)	73.2 [72.0] (52.2–98.4)	−0.01	0.401
	C	75.0 [77.4] (55.0–85.4)	77.9 [79.6] (54.6–101.2)	76.6 [78.5] (57.0–96.7)	0.02	0.09
Muscle Mass, %	B	42.6 [37.7] (25.7–144.6)	38.8 [36.9] (27.3–64.5)	38.2 [35.6] (24.2–79.9)	−0.05	0.20
	C	45.2 [44.8] (25.3–98.1)	47.3 [44.6] (19.5–120.7)	45.6 [42.0] (23.5–81.3)	0.01	0.69
ECM/BCM Index, %	B	1.6 [1.6] (−0.4–3.5)	1.6 [1.5] (0.4–3.5)	1.7 [1.5] (0.3–4.0)	0.00	0.82
	C	1.3 [1.2] (0.3–3.8)	1.3 [1.3] (−0.1–3.7)	1.3 [1.2] (0.2–3.7)	0.00	0.69

All values are expressed as median [median] (range), or percentage as indicated

T0: at start of HPN; T1: 60 days after the start of HPN; T2: 90 days after the start of HPN

*BIA* Bioelectrical impedance analysis, *PG-SGA* Patient-generated subjective global assessment; Category A = well-nourished; Category B = suspected malnutrition or moderate malnutrition; Category C = severely malnourished; *NA* Not applicable, *PS* Performance status, *mGPS* Modified Glasgow prognostic score

**Fig. 1:**
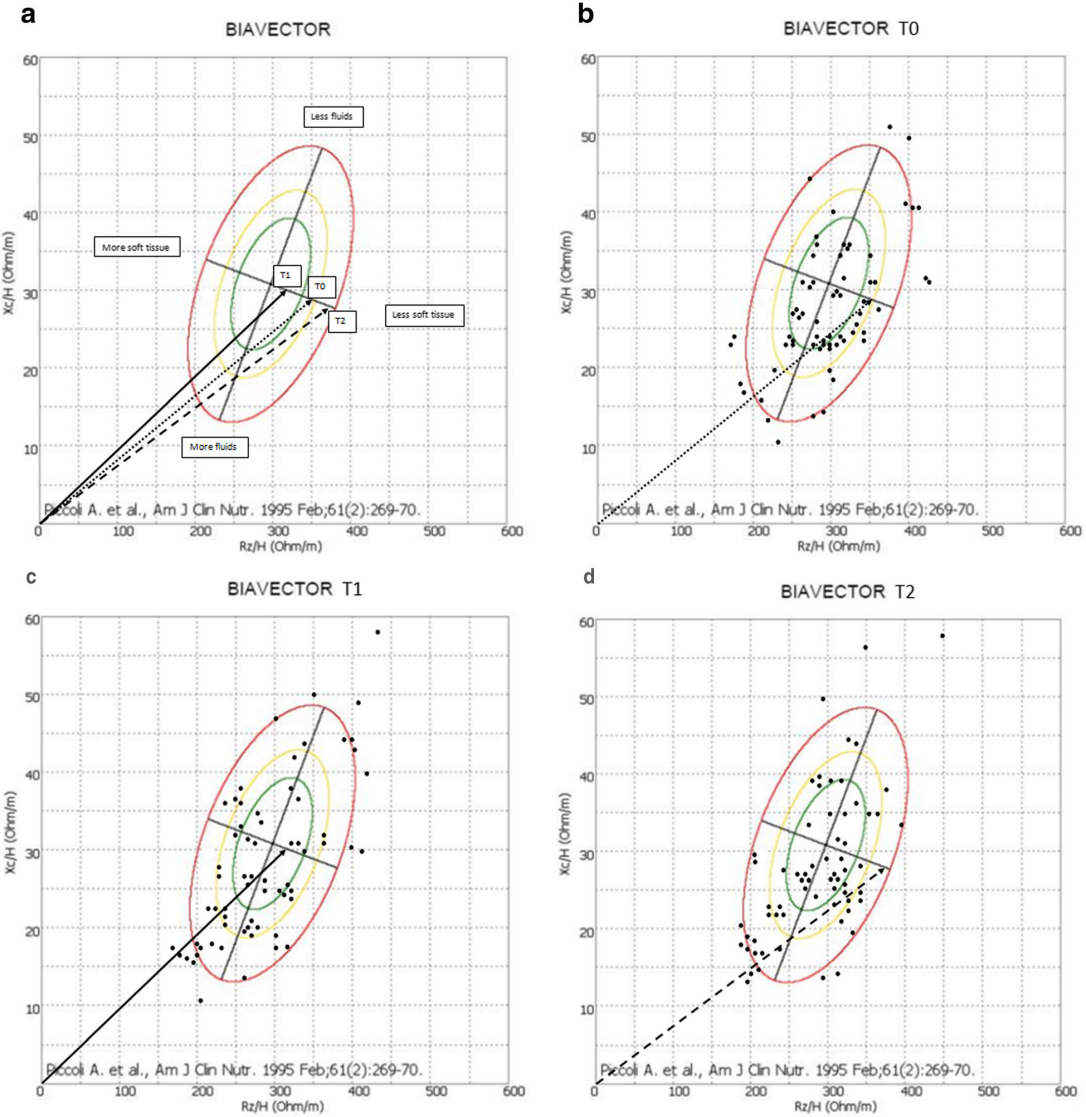
**a** BIAVECTOR at T0, T1, and T2. BIA, bioelectrical impedance analysis; T0, baseline, T1 60 and T2 90 days after the start of home parenteral nutrition; XC, reactance; RZ, resistance, H, height. **b** BIAVECTOR at the start of HPN (T0). BIA, bioelectrical impedance analysis; HPN, home parenteral nutrition; XC, reactance; RZ, resistance, H, height. **c** BIAVECTOR 60 days after the start of HPN (T1). BIA, bioelectrical impedance analysis; HPN, home parenteral nutrition; XC, reactance; RZ, resistance, H, height. **d** BIAVECTOR 90 days after the start of HPN (T2). BIA, bioelectrical impedance analysis; HPN, home parenteral nutrition; XC, reactance; RZ, resistance, H, height

BIVA summary plot for T0, T1, and T2 is shown in Fig. 1, and detailed distribution at each timepoint are shown in Fig. 1b, c and d. At T0, patients presented with altered body composition with reduced muscle mass and increased total body water. At T1, body composition improved with reduction in water and increase in soft tissue, and at T2 body composition declined. Distribution across all quadrants is seen in Fig. 1b, c and d, signifying a wide variety in patient status and disease progression.

### Survival

**Fig. 2:**
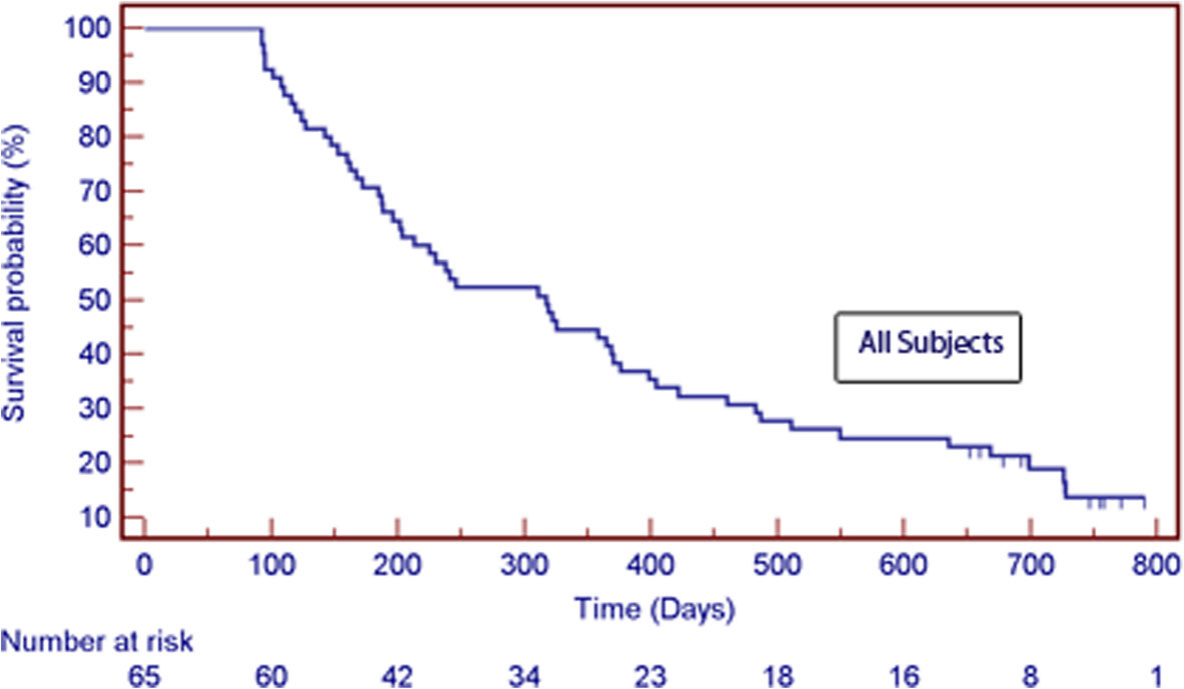
Kaplan-Meier overall survival curve

All patients were alive at T2, and at the time of analysis, 11 of the 65 patients enrolled were still alive. No patients were lost to follow-up. Figure 2 details overall survival probability, with median overall survival 317 days [range 92–790; 95% CI 219–415]. No HPN-related mortality occurred. Overall, 28 patients (43.1%) survived one-year or greater, and five patients (7.7%) survived 2 years or greater. However considering the 6-month enrollment window and the data analysis cut-off date, only 23 of the 65 patients enrolled could have potentially reached 2-year survival therefore the 2-year survival rate in eligible patients was 21.7%.

Table 3 shows BIA variables associated with survival according to Cox regression analysis. XC, RZ, and PA were significantly associated with survival at T0, T1, and T2, respectively. Among the patient variables, only PG-SGA category A at T2 (SE 1.510, *P* 0.014, 95% CI 0.001–0.465) was associated with survival. This result was confirmed by ROC curve (SE 0.076, *P* 0.006, 95% CI 0.614–0.911) also in terms of sensibility (80%), specificity (59%), and accuracy (76%).

**Table 3:** Cox regression analysis of BIA variables associated with survival

Covariate	SE	*P*	95% CI
T0			
Reactance	0.036	**0.02**	1.012–1.167
Resistance	0.118	0.05	0.630–1.001
Phase angle	0.555	0.10	0.135–1.189
T1			
Reactance	0.127	0.66	0.736–1.212
Resistance	0.009	**0.04**	1.001–1.037
Phase angle	1.134	0.44	0.260–22.161
T2			
Reactance	0.133	0.06	0.989–1.663
Resistance	0.017	0.67	0.959–1.027
Phase angle	1.470	**0.05**	1.130–1.591

T0: at start of HPN; T1: 60 days after the start of HPN; T2: 90 days after the start of HPN

*BIA* Bioelectrical impedance analysis, *SE* Standard error, *CI* Confidence interval

**Fig. 3:**
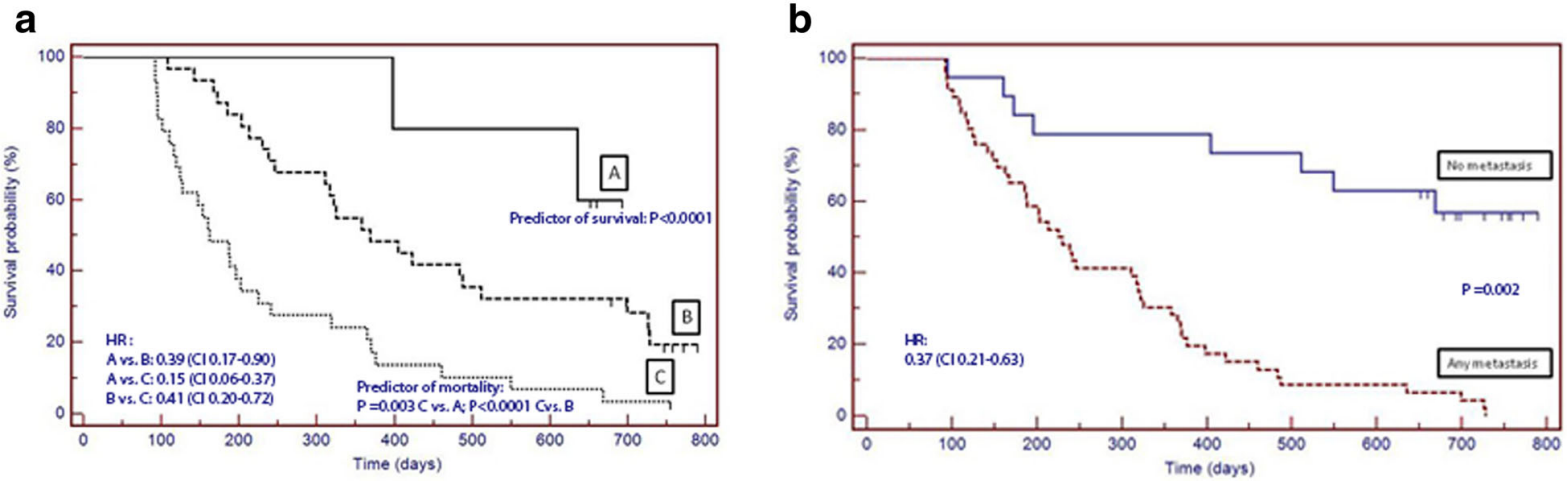
**a** Log rank test Survival curve for PG-SGA at T2. PG-SGA category A at T2 was a predictor of survival (median duration days: 652; CI 635–660; *P* < 0.0001); PG-SGA category C (median duration days: 162; CI 102–222) was a predictor of mortality vs. both A and B categories (*P* = 0.003 and *P* < 0.0001, respectively). The HR for PG-SGA category as a predictor of survival for category A vs. B was 0.39 (CI 0.17–0.90); for category A vs. C was 0.15 (CI 0.06–0.37), and for category B vs. C was 0.41 (CI 0.20–0.72). PG-SGA, Patient-Generated Subjective Global Assessment; Category A = well-nourished; Category B = suspected malnutrition or moderate malnutrition; Category C = severely malnourished; T2, 90 days after the start of home parenteral nutrition. **b** Log-rank test Survival curve for Metastasis*.* No metastasis (median duration days: 487; CI 88–894) vs. any metastasis (median duration days: 213; CI 163–263) was a predictor of survival (*P* = 0.002). The HR for no metastasis as a predictor of survival was 0.37 (CI 0.21–0.63)

At T2, the comparison of survival between curves assessed by log-rank test showed that PG-SGA category A (median duration days: 652; CI 635–660) was a predictor of survival (*P* < 0.0001); conversely, the PG-SGA category C (median duration days: 162; CI 102–222) was a predictor of mortality vs. both A and B categories (*P* = 0.003 and *P* < 0.0001, respectively) (Fig. 3). Assessing PG-SGA category at T2 as a predictor of survival, the HR for category A vs. B was 0.39 (CI 0.17–0.90); for category A vs. C was 0.15 (CI 0.06–0.37), and for category B vs. C was 0.41 (CI 0.20–0.72). The proportion of patients with improvements, declines, or no change in PG-SGA from T0 to T2 and the corresponding survival time is shown in Table 4. In rank order, patients who improved from category B to A had the longest median survival time, and patients who declined from category B to C had the shortest median survival time.

**Table 4:** Survival by change in PG-SGA from T0 to T2

	N (%)	Survival Median (range)
Improved		
B to A	5 (7.7%)	652 (398–693)
C to B	15 (23.1%)	311 (108–772)
Stayed the Same		
B to B	16 (24.6%)	452 (230–790)
C to C	19 (29.2%)	162 (92–765)
Declined		
B to C	10 (15.4%)	156 (92–376)

*PG-SGA* Patient-generated subjective global assessment, Category A = well-nourished; Category B = suspected malnutrition or moderate malnutrition; Category C = severely malnourished; T0 = baseline, T2 = 90 days after the start of home parenteral nutrition

The comparison of survival between curves showed that no metastasis (median duration days: 487; CI 88–894) vs. any metastasis (median duration days: 213; CI 163–263) was also a predictor of survival (*P* = 0.002) (Fig. 3b). Assessing no metastasis as a predictor of survival, the HR was 0.37 (CI 0.21–0.63).

## Discussion

Improving nutritional status is critical in cancer patients: treating malnutrition has been shown to improve symptoms including fatigue and appetite loss [42, 43]; improve the effectiveness of chemotherapy by reducing the likelihood of toxicity [17]; improve patient QoL [42–45]; and improve survival [16, 18].

In the current study, HPN not only provided a source of calories but also was associated with an increased oral intake with a 50% increase in oral calorie and protein intake after 90 days, possibly due to improved patient appetite and decreased side effects of the disease and treatments. This is consistent with previous studies investigating HPN in cancer patients, which reported a decrease in fatigue and appetite loss after 3 months of HPN [42, 43], as well as an association between HPN and improved nutritional status, QoL, and KPS [42].

Several studies evaluating patients undergoing chemotherapy found that not only was sarcopenia (i.e., loss of lean body mass) an independent risk factor for dose-limiting toxicities, hospitalization and survival, but patients who were malnourished at the start of treatment experienced a further nutritional decline during chemotherapy [31, 46]. Considering 100% of the advanced cancer patients enrolled in the current study were undergoing chemotherapy during the study period, the improvement in nutritional status is especially noteworthy.

The study supports the utility of BIA in the management of cancer patients. BIA may be useful for an accurate evaluation of effects of HPN over time [47]. Use of weight alone to assess effects of PN can be of limited value due to several confounding factors (e.g., edema, ascites, fluid retention), but BIA can provide valuable data on changes in body compartments, which can help clinicians more accurately interpret changes in weight. Protein loss is a challenge to reverse, and early PN seems to stabilize protein levels [9]. In the current study, HPN not only slowed the weight loss expected in the patient population, but patients experienced significant increases in weight as well as in BCM and a decrease in TBW. Considering this decrease in TBW, the increase in weight patients experienced cannot be simply attributed to an increase in body water.

BIA has also shown utility as a prognostic tool in survival for cancer patients receiving chemotherapy [47]. In the current study, there was an association between survival and reactance at baseline, potentially indicating intact cell membranes; an association with resistance at 60 days after the start of HPN; and with phase angle at 90 days after the start of HPN. BIA results vary based on age, gender and disease state [29], and although not all variables showed a correlation at all time points, longitudinal assessment of BIA can be a useful addition to provide better management of an oncology patient. Several previous studies have found no linear relationship between BIA variables and survival time [26, 32, 48], and one limitation of BIA noted is high interpatient variability [47]. Nevertheless, with interpretation in the context of an individual patient, BIA may offer a convenient, validated, non-invasive method of assessing nutritional status and survival [47].

Subjective assessment of nutritional status is commonly used to supplement objective measures, and PG-SGA has been shown to be effective in assessing nutritional status in cancer patients [49, 50]. At the initiation of the study, all patients were rated as moderately to severely malnourished. After 90 days of HPN, over 30% of patients experienced an improvement in PG-SGA assessment, with 5 patients reaching well-nourished status. The results of this study are consistent with previous research showing not only the utility of PG-SGA to assess nutritional risk, but also its value as a predictor of mortality in cancer patients [50]. In the current study, survival was correlated with PG-SGA rating at 90 days, with patients rated as poorly nourished having the lowest probability of survival. Patients who improved to a rating of well-nourished had the longest median survival in rank order, followed by patients who were consistently rated moderately malnourished at baseline and at 90 days. Shortest median survival times were seen in patients who declined from moderately malnourished to severely malnourished followed by patients who were consistently rated severely malnourished at baseline and at 90 days. These results highlight the impact of HPN in improving nutritional status, which can lead to improved survival. They also reinforce the importance of intervening with HPN in cancer patients before they reach severe malnourishment. Early intervention with HPN is reinforced by the study results, as patients who began HPN with PG-SGA rating of B experienced more significant improvements in clinical and BIA parameters than patients who began HPN with PG-SGA rating of C.

HPN can improve patient performance status, as demonstrated by the increase in KPS after 90 days of HPN seen in this study. There was a median increase in KPS of 10 after 90 days of HPN, and the proportion of patients with KPS > 70 increased by 16%. Functional status is an important factor in oncology patient QoL; moving from a KPS of 70 to 80 means the difference in being able to carry out normal activities with no special care needed. In addition to the impact on daily living, a higher KPS has been associated with better survival in cancer patients [16, 24].

Evidence shows systemic inflammatory response is associated with weight loss, loss of muscle tissue, and decreased functional status, and that inflammation scales can identify risk in cancer patients. [24, 38] The prognostic value of mGPS in cancer patients has been shown in multiple studies [51, 52]. Proctor et al. noted an association between an mGPS of 2, (i.e., the worst value from a prognostic point of view) and a 160% reduction in survival, regardless of tumor site [52]. In the current study, there was a 32% reduction in the proportion of patients categorized as mGPS of 2 after 90 days of HPN.

Over 70% of patients enrolled in the study had metastases. Research previously conducted showed that survival of cancer patients on HPN was affected by tumor spread [24], and the current study supports this finding, with metastasis as a predictor of survival.

### Strengths and limitations of the study

The strength of this study is that it prospectively enrolled a population of adult patients with many similar characteristics who all: (1) had solid tumors of advanced stages; (2) were malnourished; (3) were outpatients; (4) were receiving chemotherapy during the course of the study; (5) were managed with daily HPN and received the same protocol of care at home. Moreover, no patients were lost to follow-up, and all patients survived through the day 90 assessment.

This study has several limitations. The most important is the lack of a comparator group to assess the effects of HPN; however, considering guidelines recommending supplemental nutrition in malnourished cancer patients receiving chemotherapy [20] it would have been ethically unacceptable to have a non-HPN control arm. The second limitation is the relatively small number of enrolled patients. However, with respect to the power of statistical analyses, a sample of 52 patients would allow detection of correlations between BIA measures and survival with 80% power and α = 0.05. Because the distribution of data for BIA measures is often skewed, to reduce the type I error rate a greater number of patients was enrolled. Also, the series of patients are heterogeneous and diverse cancers are rather different in terms of nutritional aspects. However, we investigated the nutritional status of this population in-depth with two well-accepted and effective scores (i.e., PG-SGA and mGPs). Third, our intention was not to assess QoL because it was already evaluated in a previous study [43]. Finally, a detailed description of the chemotherapy and radiotherapy regimens adopted in the different sites and stages of the tumors as well as the associated toxicity was beyond the aims of this paper.

## Conclusions

To the best of our knowledge, this is the first longitudinal study investigating the impact of HPN in cancer patients receiving chemotherapy by an evaluation of body composition using BIA measures. Our study showed that malnourished patients with advanced cancer experienced significantly improved body weight, BMI, oral calorie and protein intake. After 90 days of HPN, patients had significantly improved nutritional status, some BIA measures, performance status and prognostic score. Moreover, the BIA measures reactance, resistance and phase angle were significantly associated with survival. Finally, survival was correlated with PG-SGA rating at 90 days, with patients rated as poorly nourished having the lowest probability of survival.

HPN may be an important therapy in oncology patients receiving chemotherapy. Nowadays, clinicians must use tools to identify patients early who are likely to benefit from HPN, and assist in ongoing management. Longitudinal use of BIA may help track the impact of HPN and disease progression, potentially contributing to a better management of global patient care.

## Abbreviations

BCM: Body cell mass
BIA: Bioelectrical impedance analysis
BIVA: Bioelectrical impedance vector analysis
BMI: Body mass index
ECM: Extracellular mass
ECOG: Eastern Cooperative Oncology Group
ECW: Extracellular water
FFM: Fat free mass
FM: Fat mass
GPS: Glasgow prognostic score
HPN: Home parenteral nutrition
IW: Intracellular water
KPS: Karnofsky performance status
mGPS: Modified Glasgow Prognostic Score
PA: Phase angle
PG-SGA: Patient-Generated Subjective Global Assessment
PN: Parenteral nutrition
QoL: Quality of life
R: Resistance
ROC: Receiver Operating Characteristic
TBW: Total body water
XC: Reactance
Z: Impedance
